# Dual SYK/JAK inhibition overcomes ibrutinib resistance in chronic lymphocytic leukemia: Cerdulatinib, but not ibrutinib, induces apoptosis of tumor cells protected by the microenvironment

**DOI:** 10.18632/oncotarget.14588

**Published:** 2017-01-10

**Authors:** Ailin Guo, Pin Lu, Greg Coffey, Pamela Conley, Anjali Pandey, Y. Lynn Wang

**Affiliations:** ^1^ Department of Pathology, Lymphoma Translational Pathology, University of Chicago, Chicago, IL, USA; ^2^ Portola Pharmaceuticals, Inc., South San Francisco, CA, USA

**Keywords:** CLL, cerdulatinib, SYK, JAK-STAT, molecularly targeted therapy

## Abstract

Ibrutinib (BTK inhibitor) has generated remarkable responses in CLL. However, the drug, to a large extent, does not cause cell death directly and does not eradicate CLL malignant clones. Inability to eradicate CLL has fostered resistance generation. Once patients become resistant, they do poorly with a median survival of 3-4 months. Novel therapeutic strategies are needed to prevent resistance, improve treatment outcome and ultimately cure the disease. Herein, we explore dual targeting of the BCR and JAK-STAT pathways with a novel single agent, cerdulatinib, which selectively inhibits both SYK (a BCR component) and JAK kinases. We demonstrated that cerdulatinib delivered potent tumor inhibition in 60 primary CLL patient samples, especially in those with poor prognostic indicators. Importantly, cerdulatinib, but not ibrutinib, is able to overcome the support of microenvironment and induces CLL cell death at clinically achievable concentrations. Notably, cerdulatinib blocked proliferation of ibrutinib-resistant primary CLL cells and of BTK^C481S^-transfected/ibrutinib-resistant lymphoma cells. These anti-tumor effects are well correlated with the inhibition of BCR and JAK-STAT signaling and downstream inhibition of the functions of AKT, ERK and NF?B. Collectively, our results show that simultaneous targeting of BCR and JAK-STAT pathways is a more effective strategy relative to single BTK inhibition.

## INTRODUCTION

Increased BCR signaling is considered one of the most important driving pathologic mechanisms leading to CLL development, progression and relapse. BCR-targeted therapies including ibrutinib (ibrutinib, a BTK inhibitor) have generated remarkable responses in treating mature B-cell malignancies including CLL[1–5]. Ibrutinib, in particular, was approved by the FDA in February 2014 as a breakthrough therapy for relapsed/refractory CLL, in July 2014 as a frontline therapy for high-risk CLL with 17p deletion and most recently in March 2016 as the first chemotherapy-free treatment for both treatment-naïve and previously treated patients.

Despite ibrutinib's anti-tumor activity across multiple lymphoid malignancies, clinical observations and ex-vivo studies including ours suggest that ibrutinib does not induce a significant degree of CLL cell death at *clinically achievable* concentrations [6–9] and thereby has a low potential to eradicate residual disease. Lack of cell death may account for the single-digit low complete response rate [10] and the persistence of circulating CLL cells beyond 12-months of ibrutinib treatment in some cases [10, 11]. The lack of effective killing provides tumor cells a window of opportunity to mutate and escape drug suppression. *BTK*^C481S^ mutation, identified by our group and others, arises commonly in patients who relapse on ibrutinib [12–14]. Although the reported frequency of overall resistance remains relatively low at < 10% [15, 16], clinical experience with ibrutinib is relatively short. It is foreseeable that the incidence of observed resistance will increase as clinical use outside clinical trials spreads over time. Besides resistance, ~25% of patients discontinue ibrutinib due to lack of tolerability or efficacy resulting in high fatality [15, 16]. Thus, there continues to be an urgent medical need for new therapeutic options.

Simultaneous targeting of multiple oncogenic pathways is a strategy to prevent and reduce the overall incidence of drug resistance and to potentially drive higher complete response rates [46]. Besides the BCR pathway, JAK-STAT represents another important tumor-promoting signaling pathway in the pathogenesis of CLL [17–20]. Numerous cytokine/chemokine stimuli released from the tissue microenvironment and CLL cells themselves promote the growth and survival of the malignant cells. The cytokines include, but are not limited to, IL-4, and IL-6, that act through cytokine receptors and JAK kinases to phosphorylate and activate STAT6 or STAT3, respectively. STAT activation subsequently up-regulates anti-apoptotic proteins MCL-1 and BCL-xL, increases cell survival and confers CLL resistance to cytotoxic agents. [21–23]

Cerdulatinib is a novel orally available, ATP-competitive, small-molecule inhibitor that demonstrates selective inhibition of SYK and JAK kinases with IC_50_ of 32 nM for SYK and 0.5-12 nM for JAKs [24]. Its selectivity in the cellular context was also demonstrated by the lack of inhibition of T-cell receptor signaling or protein kinase C signaling in whole blood [24]. In a mouse model, it blocks B-cell activation and alleviates splenomegaly induced by chronic BCR stimulation [24]. In human lymphoma cell lines and primary lymphoma cells, cerdulatinib demonstrates broad anti-tumor activity in diffuse large B-cell lymphoma including GCB and ABC tumor cells with ibrutinib-resistant MYD88 or CARD11 mutations [25]. In CLL, we showed that cerdulatinib blocks proliferation of *BTK*^C48S^-bearing ibrutinib-resistant cells isolated from an ibrutinib-relapsed patient [14]. Herein, we further investigate the effects of dual SYK and JAK inhibition in a collection of 60 CLL patient samples in comparison to ibrutinib. We demonstrate that cerdulatinib has additional anti-tumor activities compared to ibrutinib and the compound shows great potential in overcoming ibrutinib resistance.

## RESULTS

### CLL is sensitive to cerdulatinib especially in cases with poor prognosis

**Figure 1:**
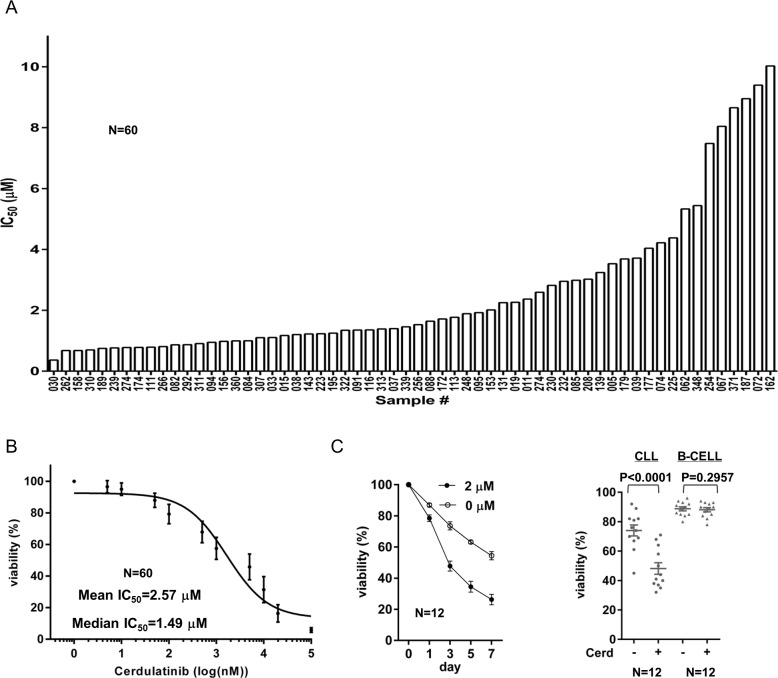
CLL are sensitive to cerdulatinib especially in cases with poor prognosis **A.** IC_50_ of cerdulatinib in 60 CLL samples. Isolated CD19^+^ cells from CLL patients were incubated with or without increasing concentrations of cerdulatinib (10^1^-10^5^ nM) for 72 hours. Viability was measured by PI staining and was normalized to the matched vehicle control for each specimen (100%). IC_50_ was then generated using the GraphPad Prism 6 program. **B.** Dose-response curve for all 60 cases. Each data point represents mean±SE of normalized viability of 60 cases at each of 11 tested concentrations. The overall IC_50_ was then generated using the GraphPad Prism 6. **C.** Left panel, Time course of viability reduction. Cells were incubated with DMSO or 2 µM cerdulatinib and cell viability was measured at the indicated time points (*N* = 12). Data points represent mean±SE. Right panel, Minimal effects of cerdulatinib in normal B cells. Cells were incubated with DMSO or 2 µM cerdulatinib. Viability of CLL cells (*N* = 12) was compared with B cells (*N* = 12) at 72 hrs following cerdulatinib addition.

We first determined sensitivity of a cohort of 60 CLL patient samples to cerdulatinib. We treated cells with serial diluted cerdulatinib and measured cell viability after 72 hrs with propidium iodide flow cytometry. IC_50_ in 60 CLL ranged from 0.37 to 10.02 µM (Figure 1A) and the dose responses for all samples are shown in Figure 1B. The average IC_50_ of cerdulatinib for the cohort was 2.57 μM and median IC_50_ was 1.49 μM. These concentrations are clinically achievable according to a recent clinical pharmacokinetic study [26]. Using 2 μM of cerdulatinib, a time course of drug treatment was performed and a time-dependent reduction in cell viability was observed (Figure 1C, left). Notably, this anti-survival effect is selective for CLL tumor cells but not for normal B cells (Figure 1C, right). We then analyzed whether cell killing by cerdulatinib differs among CLL subgroups stratified by known prognostic factors. We found that CLLs with unmutated IGHV versus mutated IGHV have lower IC_50_s and thus were more sensitive to cerdulatinib (*P* = 0.0395, Supplementary Figure 1A). CLLs with high or intermediate risk cytogenetic abnormalities including del (11q)/ trisomy 12/del(17p) were also more sensitive to cerdulatinib than those with low risk features including del (13q) or normal cytogenetics (Supplementary Figure 1B). Although there was a trend for ZAP70 positive cases to be more sensitive to cerdulatinib, the difference between the ZAP70 positive or negative subgroups did not reach statistical significance (Supplementary Figure 1C). Meanwhile, cerdulatinib sensitivity did not differ among samples from patients with different sex, different Rai stage, or different treatment status (treated vs untreated) (data not shown). Overall, we found that CLL cells are sensitive to cerdulatinib, especially in cases with poor prognosis by IGHV and cytogenetics.

### Cerdulatinib induces apoptosis in association with MCL-1 down-regulation and PARP cleavage

**Figure 2:**
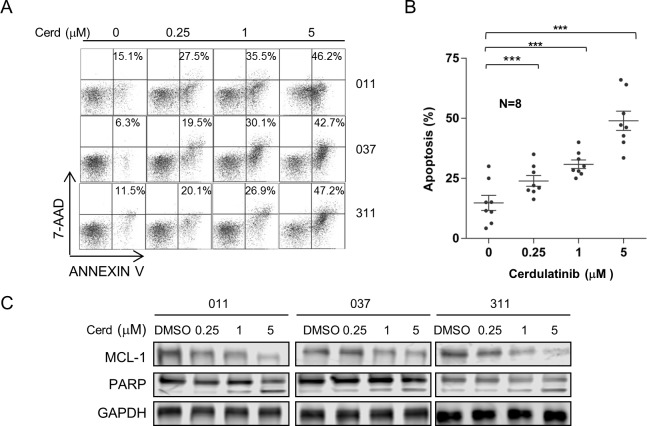
Cerdulatinib induces apoptosis in CLL in association with MCL-1 down-regulation and PARP cleavage **A.** Cerdulatinib induces apoptosis. Apoptosis was assessed by annexin V/7-AAD staining following cerdulatinib treatment for 48 hrs. Three representative cases are shown. The percentage of early apoptotic annexin-Vhi/7AAD low population in the bottom right quadrant is indicated. **B.** Dose response of 8 CLL samples at indicated concentrations of cerdulatinib post 48 hr of treatment. Data presented represent mean ± SE of apoptosis. ***, *P* < 0.001. **C.** Immunoblots of MCL-1 and PARP. Following cerdulatinib treatment for 48 hrs at indicated concentrations, MCL-1 and PARP1 cleavage were measured by Western blot in whole cell lysates. GAPDH was included as the loading control.

We next investigated if apoptosis induction is one of the mechanisms of CLL cytotoxicity induced by cerdulatinib. CLL cells were treated with different concentrations of cerdulatinib and apoptosis events were measured with Annexin V/7-AAD staining. Results of three representative cases are shown in Figure 2A and aggregate results of eight cases are shown in Figure 2B. Dose-dependent apoptosis was observed in all CLL samples tested. Furthermore, the anti-apoptotic protein MCL-1 was reduced by cerdulatinib in a dose-dependent fashion that was accompanied by dose-dependent increases of PARP cleavage (Figure 2C). Overall, the data show that cerdulatinib reduces CLL survival through the induction of apoptosis.

### Cerdulatinib, but not ibrutinib, is able to overcome the support of the microenvironment and induce CLL cell death

**Figure 3:**
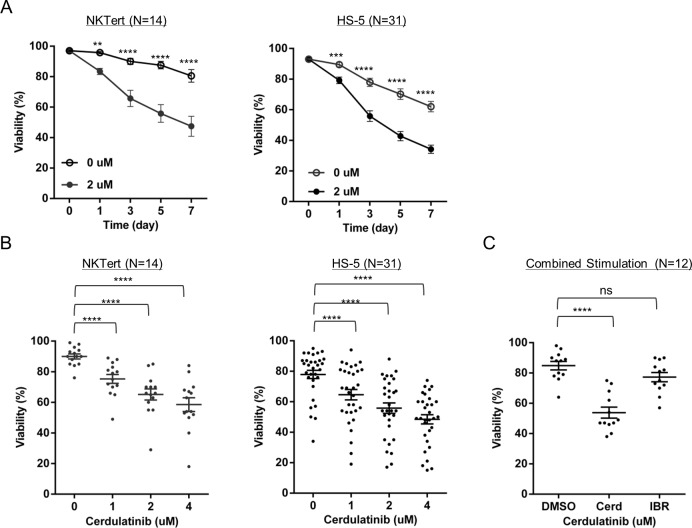
Cerdulatinib, but not ibrutinib, is able to overcome the support of the microenvironment and induces CLL cell death **A.** Time course of cell viability following 2 µM cerdulatinb treatment in the presence or absence of stromal NKTert cells (*N* = 14) (Left panel) or stromal HS-5 cells (*N* = 31) (Right panel). **B.** Dose-titration of cerdulatinib treatment (72 hrs) in the presence of stromal NKTert cells (*N* = 14) or HS-5 cells (*N* = 31). **C.** Effect of 2 µM cerdulatinib or 0.5 µM ibrutinib on CLL viability in the presence of 10 ng/mL IL4, 1µg/mLl CD40L, and 10µg/mL plate-bound αIgM. Data in A-C were analyzed by ANOVA test. **, *P* < 0.01; ***, *P* < 0.001; and ****, *P* < 0.0001. ns, not significant.

Survival of CLL tumor cells *in vivo* is heavily dependent upon survival factors from its microenvironment. Cell-to-cell contact, as well as soluble cytokines and chemokines, promote CLL survival/proliferation and protect tumors cells from killing by anti-tumor agents [21, 27–30]. In order to determine whether cerdulatinib is effective against CLL in the presence of microenvironmental support, we first tested the effects of cerdulatinib in two *in vitro* CLL co-culture models mimicking the *in vivo* microenvironment. Addition of 2μM cerdulatinib significantly reduced CLL cell viability throughout the 7-day course, even when cells were co-cultured over either NKTert or HS-5, human bone marrow stromal cell lines (Figure 3A). The anti-survival effect became more pronounced as the dose of cerdulatinib was escalated from 1 to 4 uM with both models (Figure 3B).

Our previous studies showed that ibrutinib, at a clinically achievable concentration of 500 nM (in human C_max_ is 408 nM [1]), had little effect on cell survival with stromal co-culture [9, 14]. We compared viabilities of cells treated with either cerdulatinib or ibrutinib in the presence of previously defined CLL survival signals. Cmax for each drug was used for such comparison (2 µM for cerdulatinib and 0.5 µM for ibrutinib). The results confirmed that ibrutinib did not affect cell viability whereas cerdulatinib consistently induced significant cytotoxicity in the presence of combined αIgM+ IL4+CD40L (Figure 3C). Taken together with the co-culture experiments, these results demonstrate that cerdulatinib is able to overcome the protective signals from the tumor microenvironment and reduce CLL cell viability under several tested conditions.

### Cerdulatinib blocks proliferation of primary CLL cells

**Figure 4:**
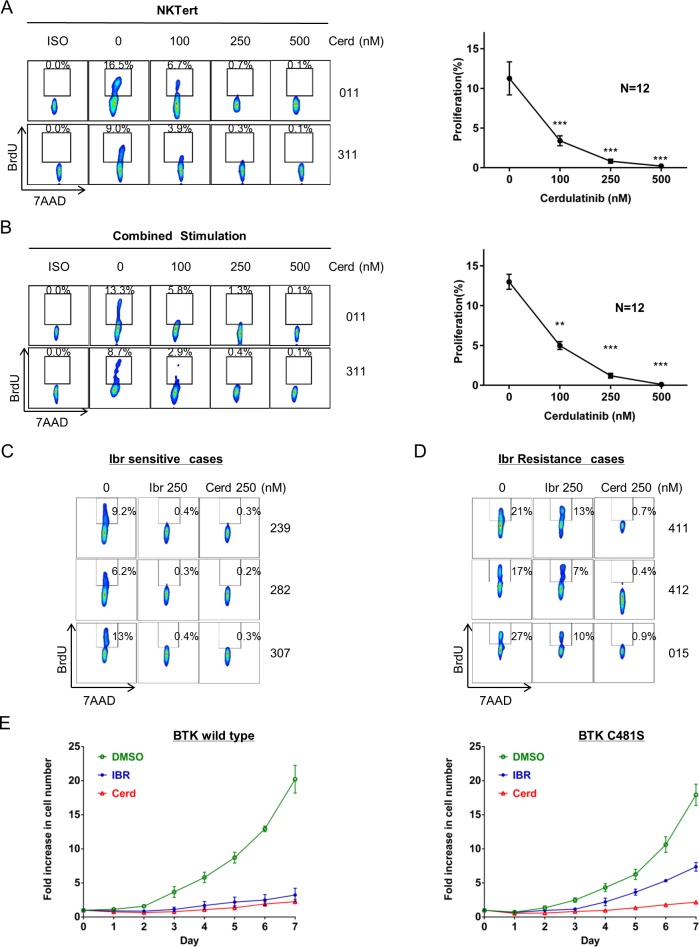
Cerdulatinib blocks proliferation of ibrutinib-sensitive and ibrutinib-resistant primary CLL cells **A.** Cell proliferation following increasing concentrations of cerdulatinb in the presence of NKTert co-cultures. BrdU incorporation was measured following 20 days of treatment. Left panel, two representative cases are shown. Right panel, aggregate data of 12 CLL cases analyzed with ANOVA test. Values in the line graph represent mean+SE. ***P < 0.001. **B.** Cell proliferation following increasing concentrations of cerdulatinb in the presence of combined stimulation. Cells were treated with plate-bound anti-IgM combined with IL4, CD40L and CpG in the presence of cerdulatinib and BrdU for 8 days followed by flow cytometric analysis for BrdU incorporation. Left panel. two representative cases are shown. Right panel, aggregate data of 12 CLL cases analyzed with ANOVA test. Values in the line graph represent mean+SE. **P < 0.01 and ***P < 0.001. **C.** Sensitivity of three ibrutinib-sensitive cases to cerdulatinib. A low in vivo achievable concentration of 250 nM was chosen to match that of ibrutinib. **D.** Sensitivity of three ibrutinib-resistant (both clinically resistant and resistant by this assay) to cerdulatinib. (C&D) Cells were treated with either 250 nM ibr or cerdulatinib in the presence of combined stimuli. BrdU incorporation was measured at day 8. **E.** Effects of ibrutinib and cerdulatinib in WT BTK-transfected TMD8 cells (Left) and in BTK^C481S^ -transfected cells (Right). 250nM of ibrutinib or cerdulatinib was added into the culture and live cell number was counted daily for 7 days. The results shown are the mean+SE of 4 replicate experiments.

We next investigated whether cerdulatinib blocks CLL proliferation in our CLL co-culture models. NKTert model has been previously optimized by our group to support proliferation of CLL so that a BrdU^+^ DNA-synthesizing CLL population can be detected [6, 9, 14]. Using this model, we have shown that one of the primary effects of ibrutinib is to block cell proliferation [6]. We then tested the effects of cerdulatinib. Two representative cases and results from an aggregate of 12 cases are shown in Figure 4A. Nearly complete blockade of CLL proliferation was achieved at 250-500 nM of cerdulatinib. These results were reproduced with a combined stimulation model in which a mixture of anti-IgM, IL4, CD40L and CpG were used to promote CLL proliferation (Figure 4B). Thus, CLL proliferation is very sensitive to cerdulatinib inhibition in both CLL proliferation models.

### Cerdulatinib blocks proliferation of both ibrutinib-sensitive and ibrutinib-resistant primary CLL cells as well as BTK^C481S^-transfected cell lines

We then compared cerdulatinib with ibrutinib side-by-side in both ibrutinib-sensitive and ibrutinib-resistant cases. Primary cells isolated from patients who responded to ibrutinib *in vivo* were treated with either ibrutinib or cerdulatinib *in vitro* under the condition of combined stimulation. Shown in Figure 4C, these cells responded equally well to either drug at a low and clinically achievable concentration. We then performed similar experiments on cells isolated from three ibrutinib-relapsed patients. These samples carry BTK mutations that confer ibrutinib resistance. Two of the patients had the known BTKC481S mutation [12, 14] and one other patient had BTK^T316A^ [31]. When these mutated cells were tested against ibrutinib and cerdulatinib, a significant number of BrdU^+^ CLL cells remained following ibrutinib treatment, whereas cerdulatinib almost completely blocked the appearance of BrdU^+^ cell populations in all three cases (Figure 4D, middle vs. right column). These experiments demonstrate that cerdulatinib not only blocks cell proliferation in ibrutinib-sensitive but also ibrutinib-resistant CLL cells.

To test whether cerdulatinib directly suppresses the growth of ibrutinib-resistant cells, we constructed and cloned both BTK^C481S^ and wild type BTK (WT) expression vectors and then transfected them into the ibrutinib-sensitive lymphoma cell line TMD8. We assessed cell growth following exposure to ibrutinib or cerdulatinib. Figure 4E showed that the growth of WT BTK -transfected TMD8 cells was similarly inhibited by both ibrutinib and cerdulatinib at 250 nM (Left panel, compare blue and red curves). However, BTK^C481S^-transfected cells were less sensitive to ibrutinib, as expected. (Figure 4E, right, blue vs green curves). Meanwhile, growth of these cells was effectively blocked by cerdulatinib (Figure 4E, right, red), similar to the block observed in WT BTK cells (Left panel). Taken together with our primary cell results (Figure 4C & 4D), these data demonstrate that cerdulatinib is capable of overcoming BTK mutation-mediated ibrutinib resistance with respect to cell growth and proliferation.

### Cerdulatinib effectively blocks BCR and JAK-STAT signaling

**Figure 5:**
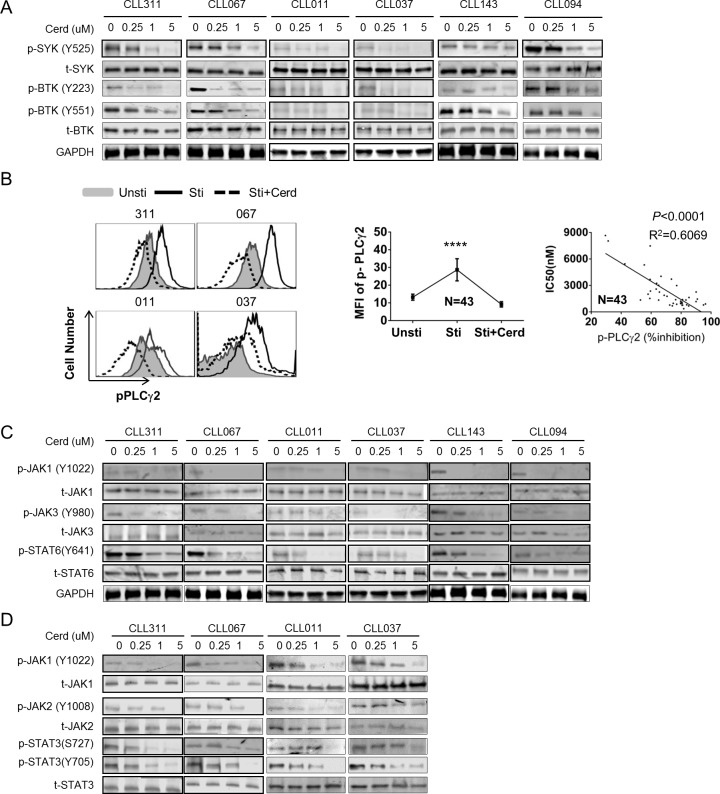
Cerdulatinib effectively blocks BCR and JAK-STAT signaling pathways **A.** Immunoblots for early BCR components SYK and BTK following cerdulatinib treatment. Freshly isolated CLL cells (*N* = 6) were treated with increasing doses of cerdulatinib in the presence of combined stimulation with soluble anti-IgM, CD40L and CpG. p-SYK, total SYK, p-BTK and total BTK were blotted in whole cell extracts. GAPDH served as a loading control. **B.** Left panel, phospho-flow assay of p-PLCγ2 (Tyr759) in 4 representative cases under different conditions. CLL cells were treated with 2 µM of cerdulatinib for 1 hr before stimulation with anti-IgM, IL-4 and CD40L. Unsti, unstimulated cells. Sti, cells stimulated with combined stimuli. Sti+Cerd, stimulated cells with exposure to 2 µM cerdulatinib. Middle panel, Mean fluorescence intensity (MFI) of p-PLCγ2 under indicated conditions (*N* = 43). Data represents mean+SE. ****P < 0.0001. Right panel, Correlation between % inhibition in pPLCγ2 and cerdulatinib IC_50_ in 43 CLL samples. Percent inhibition of pPLCγ2 MFI is defined as [(MFI_sti_ - MFI_sti+cerd_)/MFI_sti_] ×100%. Data were analyzed using Spearman correlation. P values and correlation coefficients are indicated. **C.** Immunoblots for JAK-STAT following IL4 stimulation and cerdulatinib treatment. Freshly isolated CLL cells (*N* = 6) were treated with increasing concentrations of cerdulatinib in the presence of combined stimulation with anti-IgM, CD40L and CpG. p-JAK1, total JAK1, p-JAK3, total JAK3, p-STAT6 and t-STAT6 were blotted in whole cell extracts. See Supplemental Figure 2 A and B for quantitative analysis of JAKs. **D.** Immunoblots for JAK-STAT following IL6 stimulation and cerdulatinib treatment. Freshly isolated CLL cells (*N* = 4) were treated with increasing doses of cerdulatinib in the presence of IL-6. P-JAK1, total JAK1, p-JAK2, total JAK2, p-STAT3 and t-STAT3 were blotted in whole cell extracts. See Supplemental Figure 2 C and D for quantitative analysis of JAKs.

To better understand the molecular mechanism of cerdulatinib's action, we examined the activities of key BCR enzymes in cerdulatinib-treated cells. *In vitro* cultured CLL cells were treated with various concentrations of cerdulatinib in the presence of combined stimulation. Phosphorylation of SYK at Tyr 525, which reflects SYK activity, was inhibited by cerdulatinib in a concentration- dependent fashion in most cases, while total SYK remained constant (Figure 5A). Similarly, BTK phosphorylation at Tyr 551 (phosphorylated by SYK [32, 33]) and Tyr 223 (BTK autophosphorylation which reflects BTK activity [32, 33]) were also inhibited effectively by cerdulatinib in a concentration-dependent manner (Figure 5A).

We then analyzed the phosphorylation of PLCγ2, a substrate of BTK, with phospho-specific flow cytometry. As shown with four representative cases (Figure 5B), phosphorylation of PLCγ2 increased dramatically upon combined stimulation (black open peak vs grey shaded peak) and this increase in phosphorylation was reduced by cerdulatinib treatment, even to a level below the baseline in some cases (Figure 5B Left, dot-outlined peak vs black open peak). Data generated from an aggregate of 43 cases show that the increase by stimulation and decrease by cerdulatinib treatment are statistically significant (Figure 5B middle). Notably, an inverse correlation can be demonstrated between cerdulatinib IC_50_ (See Figure 1A) and degree of p-PLCγ2 inhibition (*n* = 43) showing that a greater inhibition of the lipase activity is correlated with a higher degree of cellular inhibition (Figure 5B right). These results on SYK, BTK and PLCγ2 indicate collectively that the anti-tumor activity of cerdulatinib correlates well with its inhibition of BCR signaling activity.

Cytokines play important roles in promoting CLL survival in bone marrow or lymph nodes through JAK-STAT pathway. The cytokines include, but not limited to, IL4 and IL6, that act through cytokine receptors and JAK kinases to phosphorylate and activate STAT3 or STAT6. STAT activation subsequently leads to the up-regulation of anti-apoptotic proteins MCL-1 and BCL-xL and increases cell survival. We thus investigated whether cerdulatinib is able to suppress the pathway activation triggered by these cytokines. CLL cells stimulated with IL4 were treated with different concentrations of cerdulatinib. Figure 5C shows that phosphorylation of JAK1, JAK3 and STAT6, components of IL-4 signal transduction, were clearly inhibited by cerdulatinib in a concentration-dependent manner, while total JAK1/3 and STAT6 remained relatively constant. When CLL cells were stimulated with IL6, phosphorylation of JAK1, JAK2 and STAT3, components of IL-6 signal transduction, were also inhibited by cerdulatinib in a concentration-dependent manner, while total proteins remained largely unchanged (Figure 5D). Together, these studies demonstrate that cerdulatinib inhibits BCR and cytokine-dependent signaling pathways in CLL.

### Inhibition of SYK and JAK by cerdulatinib translates to downstream inhibition of AKT and ERK

**Figure 6:**
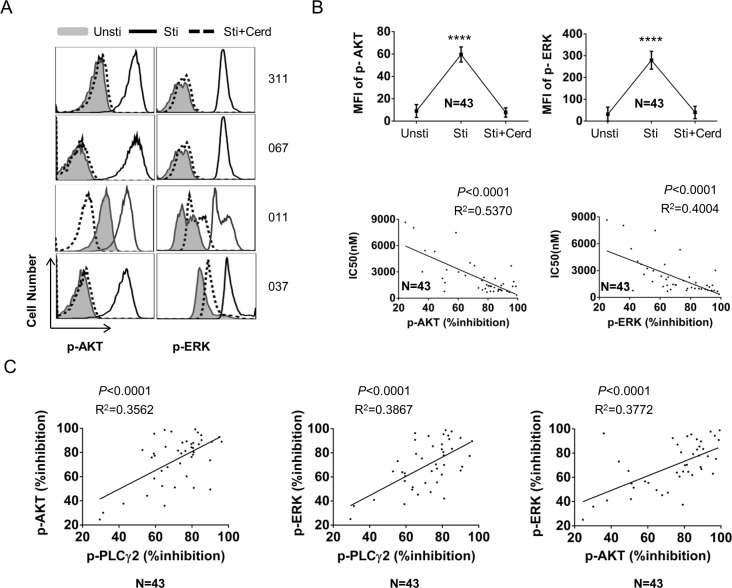
Activity of cerdulatinib is transduced to inhibition of AKT and ERK **A.** Phospho-flow assays of p-AKT (Ser473) and p-ERK (Thr202/Tyr204), in 4 representative cases under different conditions. CLL cells were treated with 2 µM of cerdulatinib for 1 hr before stimulation with soluble anti-IgM, IL-4 and CD40L. Unsti, unstimulated cells. Sti, cells stimulated with combined stimuli. Sti+Cerd, stimulated cells with exposure to 2 µM cerdulatinib. **B.** Top panels, Mean fluorescence intensity (MFI) of p-AKT or p-ERK under indicated conditions (*N* = 43). Data represents mean+SE. ****P < 0.0001. Bottom panels, correlation between % inhibition in p-AKT or p-ERK and cerdulatinib IC_50_ in 43 CLL samples. **C.** Correlation between % inhibition of different signaling parameters. Percent inhibition of phospho-proteins is defined as [(MFI_sti_ - MFI_sti+cerd_)/MFI_sti_] ×100%. Data were analyzed using Spearman correlation. P values and correlation coefficients are indicated.

BCR or cytokine signaling directly or indirectly leads to downstream activation of AKT and ERK [6, 21, 24]. We thus examined whether activity of AKT and ERK was inhibited by cerdulatinib. Phosphorylation of AKT and ERK was assessed by phospho-flow, a sensitive and quantitative assay. As illustrated with four representative cases in Figure 6A, activity of AKT and ERK was significantly increased by combined stimulation (black open peak vs grey shaded peak), and this increase was significantly reduced or even completely abolished by cerdulatinib (dot-outlined peak vs black open peak). Data derived from 43 CLL cases were summarized (Figure 6B top). Similar to PLCγ2, the degree of both p-AKT and p-ERK inhibition is inversely correlated with the IC_50_ of cerdulatinib in 43 CLL samples suggesting the anti-tumor effect of cerdulatinib relates to its inhibition of distal signal transducers AKT and ERK (Figure 6B bottom). We also analyzed the quantitative relationship between the degree of p-PLCγ2, p-AKT and p-ERK inhibition. Statistical analysis revealed a significant linear inter-correlation between all three parameters suggesting the three signaling molecules are interrelated in the same network that is inhibited by cerdulatinib (Figure 6C).

### Cerdulatinib inhibits the activity of NF-kB pathway

**Figure 7:**
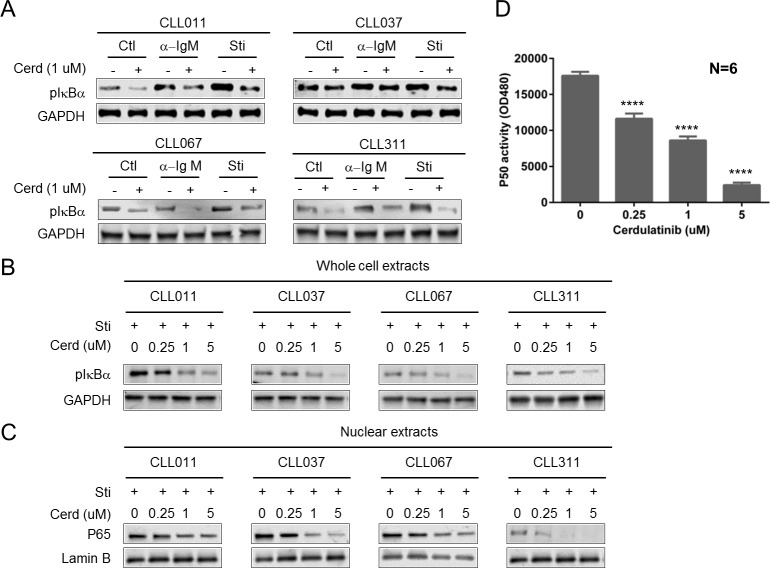
Cerdulatinib inhibits the activity of NF-κB pathway **A.** Phosphorylation of IκBα following cerdulatinib treatment. CLL cells were stimulated with either plate-bound αIgM alone or combined IL4+CD40L+plate-bound αIgM stimuli, and treated with 2 µM cerdulatnib for 24 hrs. Whole Cell lysates were immunoblotted for p-IκBα and GAPDH as the loading control. Four representative blots are shown. **B.** Reduction in p-IκBα is dose-dependent on cerdulatinib. CLL cells were stimulated with combined IL4+CD40L+plate-bound αIgM stimuli. **C.** Cerdulatinib decreased nuclear P65. Nuclear extracts were prepared from each sample and were immunoblotted for P65 and lamin B, a nuclear marker. **D.** DNA binding activity of NFκB subunit p50. Nuclear extracts were prepared from each sample. P50 activity was measured by ELISA plates coated with oligonucleotides containing the NF-kB consensus sequence (59-GGGACTTTCC-39). Concentration of p50 was determined by comparing samples with a standard curve of purified p50 protein, results represent mean±SE of 6 CLL samples. ****P < 0.001.

BCR signaling ultimately results in NFκB activation. BCR-mediated activation of IKKβ phosphorylates IkBα leading to its degradation and subsequent release of p65 and p50 from an inhibitory complex. Activated P65/P50 then translocates to the nucleus and activates its target genes essential for cell activation, survival, and proliferation[34]. We first interrogated the phosphorylation of IκBα in whole cell lysate following cerdulatinib treatment. Figure 7A shows that stimulation of CLL cells (*n* = 4) with either αIgM or combined stimuli (sti) induced IκBα phosphorylation in three of four samples tested (CLL011, CLL067 and CLL311), while cerdulatinib treatment reduced IκBα phosphorylation in all samples. Figure 7B further demonstrates that inhibition of IκBα phosphorylation occurred in a concentration-dependent manner in whole cell lysates with concomitant reduction of *nuclear* p65 (Figure 7B & 7C). Together, these results suggest that p65 was trapped in the cytoplasmic inhibitory complex with unphosphorylated IkBα upon cerdulatinib treatment. We then further interrogated the DNA binding activity of NFkB subunit p50 using an ELISA assay. Figure 7D shows the dose-dependent reduction of p50 activity following cerdulatinib treatment. Taken together, multiple experiments clearly demonstrate that cerdulatinib inhibits the function of the NF-kB pathway from cytoplasm to nucleus.

## DISCUSSION

In this report, we evaluated the efficacy of cerdulatinib in a cohort of 60 CLL patient samples and found that the compound is effective against CLL cells at clinically achievable low concentrations that generate little toxicity in normal B cells (Figure 1). We also found that CLL cases with unmutated IGHV are more sensitive to cerdulatinib, which is consistent with our previous results that this subtype of CLL has higher intrinsic BCR activity rendering them more vulnerable to BCR-targeted therapy [9]. In addition, we found that high-risk CLL by cytogenetic abnormalities including cases with del (11q) and del (17p) are more sensitive to cerdulatinib. Although the results are statistically significant, the findings will need to be confirmed with a larger number of cases.

Notably, through a side-by-side comparison, cerdulatinib, but not ibrutinib, induced apoptosis of tumor cells in the presence of stromal co-culture and in the presence of combined micro-environmental stimuli including IL-4, CD40L and anti-IgM (Figure 3). These results are in line with our previous in vitro study as well as in vivo study that demonstrated lack of significant apoptosis induction by ibrutinib in patients receiving the therapy [6, 9]. Apoptosis induction by cerdulatinib is remarkable as the class of BCR-directed agents display limited capacity of apoptosis induction ( < 20%) in the presence of stromal protection at clinically achievable concentrations. Besides ibrutinib, these include dasatinib [35, 36], SYK inhibitors [37–40]. Several published reports that observed >20% apoptosis with these agents were conducted in the absence of stromal support and/or at clinically irrelevant high concentrations when the study drug hits many unintended molecular targets. Clinical observations also seem to support lack of apoptosis, as complete responses to BCR-directed agents are rare [41–44].

These results are also similar to what we have previously found in DLBCL. Cerdulatinib induces apoptosis in addition to causing cell cycle arrest in primary tumor cells and cell lines. The apoptosis induction feature is again superior compared to several drugs in the class of BCR-targeted therapies such as LYN and SYK inhibitors [35, 37]. Further, cerdulatinib kills DLBCL cell lines that carry *MYD88*, *CARD11* or *A20* mutations which imparts resistance to ibrutinib [25].

The ability of cerdulatinib to induce CLL apoptosis was demonstrated in a recent study by Blunt et al [45]. It has been shown that: 1) Cerdulatinib inhibits BCR-induced signaling; 2) Cerdulatinib inhibits chemokine secretion in response to BCR ligation and co-culture with nurse-like cells; 3) IL-4 mediated signalling and increased IgM expression are inhibited by cerdulatinib; 4) Cerdulatinib reduces CLL cell viability in a concentration, time and caspase dependent manner; 5) Cerdulatinib reduces cell viability in the presence of microenvironmental support and 6) Cerdulatinib and venetoclax synergize to induces substantial apoptosis in the presence of IL- 4/CD40L. Our investigation largely confirmed these findings. However, we further advanced our understanding of mechanisms of action by showing: 1) Cerdulatinib, but not ibrutinib, is able to overcome the support of the microenvironment and induce CLL cell death; 2) Cerdulatinib blocks *proliferation* of primary CLL cells in our *unique* model that promotes CLL proliferation; 3) Cerdulatinib blocks proliferation not only in ibrutinib-sensitive CLL cells, but also in ibrutinib-resistant primary cells harboring the known BTK mutations; 4) Cerdulatinib blocks growth of lymphoma cells rendered ibrutinib-resistant by the introduction of BTK^C481S^; 5) Cerdulatinib effectively inhibits JAK-STAT signaling (this was shown not only with IL-4 signaling but also with IL-6 signaling); 6) Cerdulatinib inhibits the activity of NF-kB pathway.

In addition to these unique findings, we tested the effects of cerdulatinib in a larger cohort of 60 CLL samples while no more than 12 samples were studied at a time for each experiment described in the published report [45]. Further, we demonstrated with stronger evidence that cerdulatinib effectively blocks BCR signaling. We showed, with 43 cases, extensive quantitative inverse correlations between cerdulatinib IC_50_ and the degree of inhibition of p-PLCγ2, p-AKT and p-ERK as well as pair-wise correlation between the degree of inhibition of p-PLCγ2, p-AKT and p-ERK. We thus demonstrated that the three BCR signaling molecules are interrelated in the same network that is inhibited by cerdulatinib. Overall, the two investigations are complementary to each other. The current study has delivered a new message showing that cerdulatinib overcomes ibrutinib resistance with underlying mechanistic insights supporting such a statement.

The pro-apoptotic activity of cerdulatinib is likely related to its ability to inhibit JAK-STAT pathway. Previous studies have shown that IL4 and IL6-induced STAT activation up-regulates anti-apoptotic proteins MCL-1 and BCL-xL, increase cell survival and confers CLL resistance to cytotoxic agents [21],[23]. Herein, we have shown that cerdulatinib effectively inhibited IL4-induced phosphorylation of JAK1/3 and STAT6. It also inhibited IL6-induced phosphorylation of JAK1/2 and STAT3 (Figure 6) with ultimate inhibition of MCL-1 and induction of PARP cleavage (Figure 2). Thus, our data are consistent with the previous cytokine studies and further suggest that cerdulatinib targets cytokine-induced signaling and antagonizes micro-environment-mediated CLL survival.

With wider use of ibrutinib the number of ibrutinib-resistant patients is likely to grow. With this comes increasing demand for alternative therapeutic agents for relapsed patients who bear very poor outcomes. The mechanisms of resistance are diverse, thus, simultaneous targeting of multiple pathways shows promise as an alternative therapy [46]. With its ability to induce apoptosis, cerdulatinib has the potential to serve as a more effective therapy for CLL and other B-cell lymphomas in general. Phase I clinical trial is ongoing. Preliminary results in patients with relapsed/refractory non-Hodgkin lymphoma and CLL displayed promising anti-tumor activity and an acceptable safety profile [47](NCT01994382). In vitro findings reported herein will be confirmed by correlative studies that will be conducted in parallel to the upcoming phase II trial of cerdulatinib in relapsed/refractory CLL patients.

## MATERIALS AND METHODS

### Healthy donor and CLL samples

Peripheral blood samples of 60 CLL patients were collected for this study and informed consents were obtained from all patients according to the Declaration of Helsinki, and approved by the Institutional Review Board of the Weill Cornell Medical College and University of Chicago. CLL diagnosis was based on the clinical and immunophenotypic criteria outlined by IWCLL criteria [48]. Patient characteristics are listed in supplemental Table 1. Patients investigated had not received any treatment for a period of at least 3 months before sample collection.

### Reagents

Ibrutinib was purchased from Selleckchem (Houston, TX, USA), cerdulatinib was provided by Portola Pharmaceuticals Inc. (South San Francisco, CA, USA), CpG (ODN2006, stimulatory CpG-ODN type B, human specific) was purchased from Invivogen (San Diego, CA, USA), IL-6 was from R&D Systems (Minneapolis, MN), and IL-4 and CD40L were from Enzo Life Sciences (Plymouth Meeting, PA, USA). Antibodies: anti-phosphorylated BTK (p-BTK) (Y223), p-IκBα (S32/36) p-STAT3 (Y705), STAT3, MCL-1, p-JAK1 (Y1022), p-JAK3 (Y980), p-STAT6 (Y641), p-STAT3 (S727), and p-JAK2 (T1007/1008) were purchased from Cell Signaling Technology (Danvers, MA, USA); anti-total BTK antibody, poly ADP-ribose polymerase (PARP), and the BrdU detection kit were from BD Biosciences (San Jose, CA, USA); anti-p65, STAT-6, STAT-3, JAK1, JAK2, JAK3 and GAPDH antibodies were purchased from Santa Cruz Biotechnology (Santa Cruz, CA, USA). For flow cytometry, FITC-anti-CD19 (clone HIB19) and PE-anti-CD5 (clone UCHT2) were purchased from eBioscience (San Diego, CA, USA). Alexa Fluor® 647-anti-p-AKT (Ser473) and Alexa Fluor® 488 anti-p-p44/42 MAPK (ERK1/2) (T202/Y204) were purchased from Cell Signaling Technology, and PE-anti-p-PLCγ2 (Y759) was purchased from BD Bioscience.

### CLL cell isolation and culture

CLL cells were purified using the Human B cell Enrichment Cocktail Kit (StemCell Technologies, Vancouver, BC, Canada) and were stained with anti-CD5/CD19 for verification of the purity, which was greater than 95% for all cases. Isolated CLL cells were cultured in RPMI-1640 with 15% fetal bovine serum (Gibco, Grand Island, NY, USA), penicillin (100 IU), and streptomycin (100 μg/mL), at a density of 1×10^7^ cells/mL in the presence or absence of 2.5 mg/mL CpG, 100 ng/mL CD40L, 10ng/mL IL-4. Anti-IgM stimulation was conducted with plate-bound anti-IgM (10 μg/mL). Specific conditions for each experiment are described in the corresponding figure legends. CLL cells were stimulated with IL-6 (10 ng/mL) to detect the phosphorylation of JAK1/JAK2 and STAT3.

### Co-culture conditions

Human bone marrow stromal cell line HS-5 was obtained from ATCC and NK-Tert (NKTert) was kindly provided by Dr. Jan A. Burger (M.D. Anderson), CLL cell and stromal cell co-culture assays were described previously [9, 14]. Briefly, stromal cells were seeded at a concentration of 5×10^4^ cells/per well in 24-well plates and were incubated for 24 hours to allow cells to adhere. CLL cells were then added to the culture at a ratio of 100:1 (5 x10^6^cells/mL) on confluent layers of stromal cells in RPMI medium. CLL cells were harvested by gentle pipetting, leaving the adherent stromal cell layer intact.

### Generation of BTK C481S and T316A mutant constructs

BTK wild type (WT) cDNA clone in pCMV6 expression vector was purchased from ORIGENE (Rockville, MD USA). BTK^C481S^ and BTK^T316A^ mutant vectors were generated using the QuikChange II Site-Directed Mutagenesis Kit (Agilent Technologies, Cedar Creek, TX, USA) following manufacturer's instructions. The identity of the mutant constructs was confirmed by Sanger sequencing.

### Cell transfection, cell count, viability and survival assay

TMD8 cells were transfected with constructs of WT BTK or BTK^C481S^ mutants using kit V, Program U-13 on Amaxa Nucleofector, according to the manufacturer's protocols (Amaxa, Cologne, Germany). After transfection, the cells were co-cultured with NKTert cells in a 24-well plate for 24 hrs for recovery. Ibrutinib, cerdulatinib and vehicle (DMSO) were then added into the transfected TMD8 cells and cellular viability was determined with Muse™ Count & Viability kit using Muse Cell Analyzer (Millipore, Hayward, CA, USA). The cell survival was determined by flow cytometry using the Annexin V/7-AAD Apoptosis Detection Kit I on freshly isolated CLL cells following manufacturer's instructions (BD Biosciences).

### Analysis of NF-κB activity

Nuclear extracts were obtained from six purified CLL samples and assayed for NF-κB activity using the Trans^AM^ p50 transcription factor assay (Active Motif, Carlsbad, CA, USA). According to the manufacturer's protocol, 2 µg of nuclear extracts were added to a 96-well plate pre-coated with oligonucleotide containing the NF-κB consensus DNA-binding site (5′-GGGACTTTCC-3′). DNA binding was then detected by p50 primary antibody followed by a horseradish peroxidase-conjugated secondary antibody.

### Statistical analysis

Student's paired t-test was used for analyzing the statistical significance between two sample groups and one-way ANOVA was used for multi-group comparison. Spearman correlation was performed for correlation studies. All statistical analyses were conducted using Graphpad Prism 6.0 (GraphPad, La Jolla, CA, USA). P values of less than 0.05 were considered statistically significant.

See supplemental materials for details on other routine procedures.

### Authorship contributions

YLW and GC formed the hypothesis. AG developed the assays, designed and performed the experiments, solved technical problems, and analyzed the data. PL analyzed the data and wrote the manuscript; GC, AP and PC contributed useful discussions and suggestions. YLW directed and coordinated the project designed the experiments, analyzed the data and wrote the manuscript.

## SUPPLEMENTARY MATERIALS FIGURES


