# Supplementary material for: Dual SYK/JAK inhibition overcomes ibrutinib resistance in chronic lymphocytic leukemia: Cerdulatinib, but not ibrutinib, induces apoptosis of tumor cells protected by the microenvironment
Source: Oncotarget. 2017 Jan 10;8(8):12953–67. doi: 10.18632/oncotarget.14588 (PMC5355069; doi:10.18632/oncotarget.14588)
Supplement: Supplementary file 1 [file oncotarget-08-12953-s001.pdf]

Dual SYK/JAK inhibition overcomes ibrutinib resistance in chronic lymphocytic leukemia: Cerdulatinib, but not ibrutinib, induces apoptosis of tumor cells protected by the microenvironment

Cell viability assay and IC $_{50}$ determination

Isolated $CD5^+/CD19^+$ cells from CLL patients were incubated with or without increasing concentrations of cerdulatinib (10^1-10^5 nM) for 72 hours and cell viability was measured by staining with 2 µg/mL propidium iodide (PI) (Molecular Probe), as described previously[1-3] . Ten thousand events in a live cell gate were counted by a FACS LSR2 (BD Biosciences) and the data was normalized to the matched vehicle control for each specimen (100%). IC_{50} was then generated using the GraphPad Prism 6 program (San Diego, CA, USA).

Measurement of apoptosis

Apoptotic cells were quantified by flow cytometry after annexin V-FITC and 7AAD labelling (BD Biosciences). Briefly, 10^5 cells were washed with ice-cold PBS and incubated for 15 min in the dark in 50 µl of binding buffer containing annexin V-FITC/PI. Apoptosis was measured as the percentage of Annexin V^{hi} cells.

Cell proliferation assays

Bromodeoxyuridine (BrdU) was added at the beginning of the 20-day co-culture with NKTert cell or the 8-day culture with combined stimulation (2.5 µg/mL CpG, 100 ng/ mL CD40L, 10 ng/ mL IL-4 and 10 µg/ mL plate-bound anti-IgM). The percentage of $BrdU^+$ cells was analyzed by flow cytometry using the BrdU Flow kit (BD Biosciences) according to the manufacturer's instructions.

Preparation of nuclear extracts

CLL cell nuclear and cytosol extracts were prepared with Nuclear Extract Kit (Active Motif, Carlsbad, CA, USA). Briefly, CLL cells (1×10^7) were harvested, washed, and suspended in 400 µL of cold buffer A with 1 × protease inhibitor cocktail II and III and 1 × phosphatase inhibitor. After 15 minutes on ice, 10% NP40 was added and lysates were vortexed for 10 seconds and centrifuged. The supernatant (cytosolic fraction) was removed and 50µL of cold buffer C was added to the pellet (nuclear fraction) and was rocked on a rotary shaker (4°C, 20 minutes). After centrifugation, protein content was determined by the colorimetric assay (Bio-Rad), and nuclear extracts were analyzed by immunoblotting.

Immunoblotting

Whole-cell extracts were prepared by lysing the fresh normal or CLL cells in RIPA buffer (50 mM Tris–HCl, pH 7.5, 150 mM NaCl, 1% Triton X-100, 0.5% sodium deoxycholate, 0.5% SDS, 2 mM EDTA, 1 × protease inhibitor cocktail II and III and 1 × phosphatase inhibitor). Proteins were separated on 4–12% NuPAGE gel (Bio-Rad), and transferred onto a PVDF membrane, incubated with the appropriate primary and secondary antibodies, and visualized with LI-COR imager (LI-COR biosciences, Lincoln, NE, USA).

Flow cytometric analyses

Cell staining for FACS analysis was done with an optimized amount of fluorochrome conjugated mAbs as described previously[1-4]. Briefly, after washing twice, 1x10^6 cells were re-suspended in 100 µL washing buffer (1×PBS, 0.5% BSA, 0.1% NaN_3) and stained with fluorochrome conjugated mAbs and incubated for 20 min at room temperature. Cells were washed twice in Wash buffer before scanning by flow cytometer. For intracellular phosphoflow analysis, freshly isolated CLL cells were immediately fixed with 2–4% paraformaldehyde and stored at −80 °C. The cryopreserved cells were thawed at room temperature and permeated with 50% methanol on ice for 4 h. 1x10^6 cells were suspended in 100 µL washing buffer and stained with fluorochrome conjugated mAbs and incubated for 20 min at room temperature. Flow cytometry was then conducted with LSR2 flow cytometer (BD Biosciences), and the data were analyzed using the FlowJo software (FLOWJO LLC, Ashland, OR, USA).

REFERENCES

1. Song Z, Lu P, Furman RR, Leonard JP, Martin P, Tyrell L, Lee FY, Knowles DM, Coleman M, Wang YL. Activities of SYK and PLCgamma2 predict apoptotic response of CLL cells to SRC tyrosine kinase inhibitor dasatinib. Clin Cancer Res. 2010; 16(2): 587-599.
2. Cheng S, Ma J, Guo A, Lu P, Leonard JP, Coleman M, Liu M, Buggy JJ, Furman RR, Wang YL. BTK inhibition targets in vivo CLL proliferation through its effects on B-cell receptor signaling activity. Leukemia. 2014; 28(3): 649-657.
3. Guo A, Lu P, Galanina N, Nabhan C, Smith SM, Coleman M, Wang YL. Heightened BTK-dependent cell proliferation in unmutated chronic lymphocytic leukemia confers increased sensitivity to ibrutinib. Oncotarget. 2016; 7(4): 4598-4610. doi: 10.18632/oncotarget.6727.
4. Cheng S, Coffey G, Zhang XH, Shaknovich R, Song Z, Lu P, Pandey A, Melnick AM, Sinha U, Wang YL. SYK inhibition and response prediction in diffuse large B-cell lymphoma. Blood. 2011; 118(24): 6342-6352.

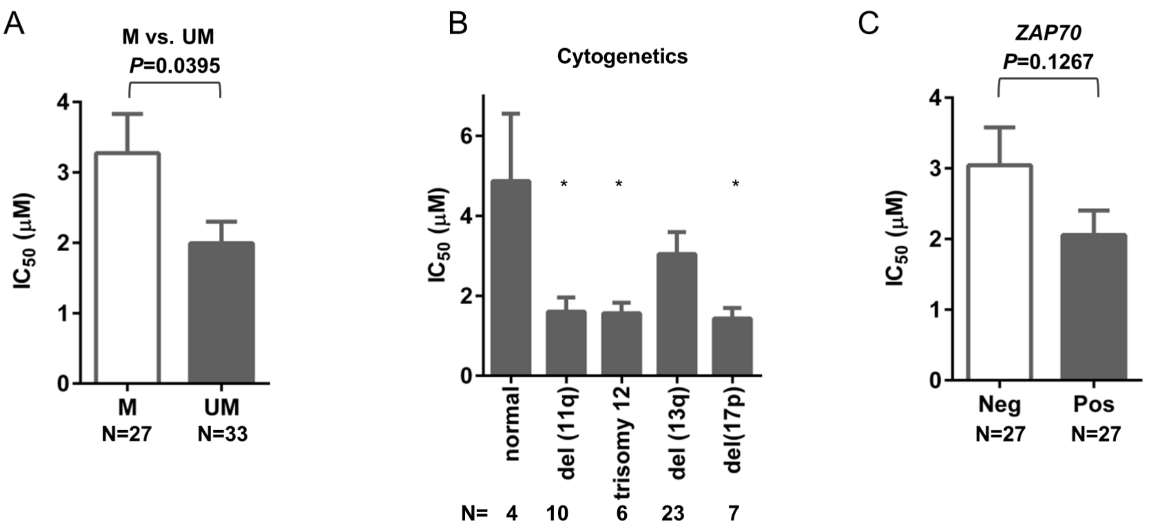

Supplementary Figure 1: Effects of cerdulatinib in CLL subgroups of known prognostic factors. (A) CLL cells with unmutated (UM) IGHV (N=33) are more sensitive to cerdulatinib than IGHV mutated (M) CLL (N=27). Data was analyzed with Student T test. Mean+SE of IC_{50} are plotted. *P*=0.0395. (B) Cerdulatinib sensitivity of CLL cells with different cytogenetic abnormalities. Case numbers for each subgroup are indicated. Data was analyzed by ANOVA test, Mean+SE of IC_{50} are plotted.*, P<0.05. (C) Cerdulatinib sensitivity of CLL with different ZAP70 status (N=27 for each subgroup). Data was analyzed with Student T test. Mean+SE of IC_{50} are plotted. *P*=0.1267.

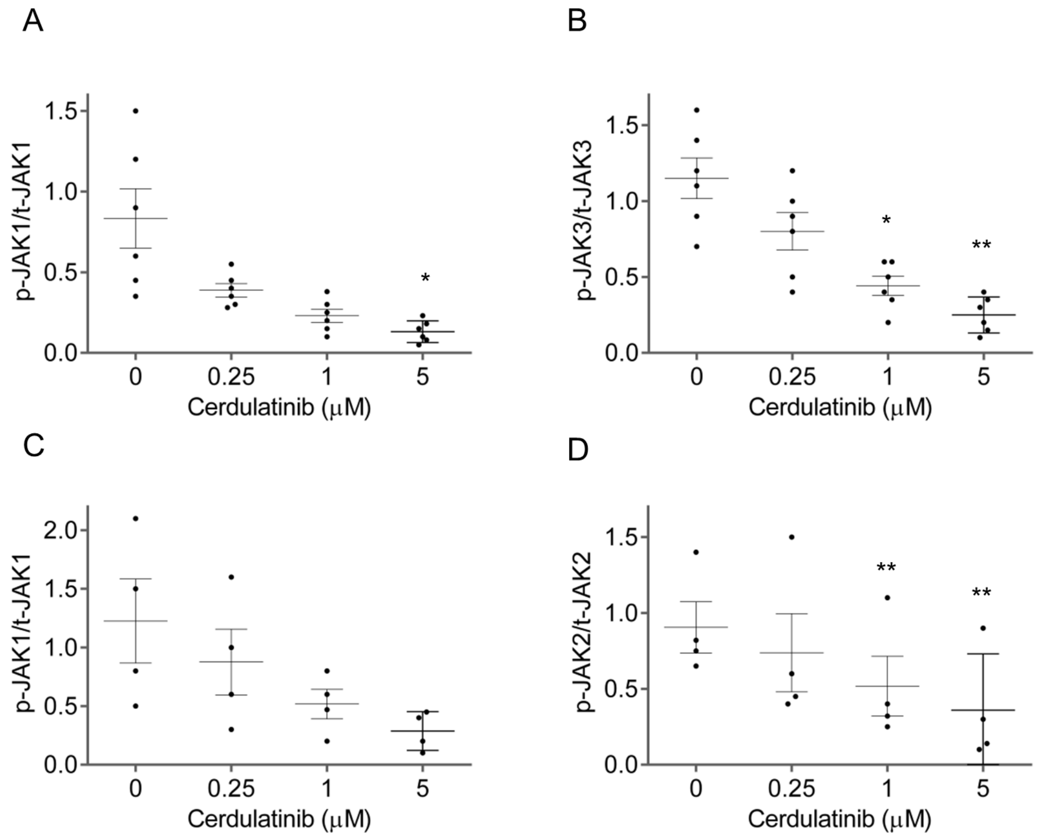

Supplementary Figure 2: Cerdulatinib effectively blocks JAK-STAT signaling pathways. (A-B) Immunoblots for JAK-STAT following IL4 stimulation and cerdulatinib treatment. Expression levels of p-JAK1at Y1022 or p-JAK3 at Y980 was quantified and normalized to total JAK1 (A) or JAK3(B), respectively. (C-D) Immunoblots for JAK-STAT following IL6 stimulation and cerdulatinib treatment. Expression levels of p-JAK1at Y1022 or p-JAK2 at Y1008 was quantified and normalized to total JAK1 (C) or JAK2(D), respectively. Paired one-way ANOVA was performed to compare the relative expression of JAKs in different concentration groups. Middle bars represent Means±SEM. *$p<0.05$, **$P< 0.01$.
